# Efficacy and safety of trifluridine/tipiracil in older and younger patients with metastatic gastric or gastroesophageal junction cancer: subgroup analysis of a randomized phase 3 study (TAGS)

**DOI:** 10.1007/s10120-021-01271-9

**Published:** 2022-01-08

**Authors:** Kohei Shitara, Toshihiko Doi, Hisashi Hosaka, Peter Thuss-Patience, Armando Santoro, Federico Longo, Ozgur Ozyilkan, Irfan Cicin, David Park, Aziz Zaanan, Carles Pericay, Mustafa Özgüroğlu, Maria Alsina, Lukas Makris, Karim A. Benhadji, David H. Ilson

**Affiliations:** 1 Department of Gastrointestinal Oncology, National Cancer Center Hospital East, Kashiwa-shi, Chiba 277-8577 Japan; 2 Department of Gastroenterology, Gunma Prefectural Cancer Center, Ota, Gunma Japan; 3 Medizinische Klinik m.S. Hämatologie, Onkologie und Tumorimmunologie, Charité-Universitätsmedizin Berlin, Berlin, Germany; 4 Department of Biomedical Sciences, Humanitas University, Via Rita Levi Montalcini 4, Pieve Emanuele, 20072 Milan, Italy; 5 IRCCS Humanitas Research Hospital, Humanitas Cancer Center, Via Manzoni 56, Rozzano, 20089 Milan, Italy; 6 Medical Oncology, Hospital Universitario Ramon y Cajal, IRYCIS, CIBERONC, Madrid, Spain; 7 Medical Oncology, Baskent University Adana Practice and Research Centre Kisla, Adana, Turkey; 8 Department of Internal Medicine, Division of Medical Oncology, School of Medicine, Trakya University, Edirne, Turkey; 9 Hematology and Oncology, St. Jude Crosson Cancer Institute/St. Joseph Heritage Healthcare, Fullerton, CA USA; 10 Department of Gastrointestinal Oncology, European Georges Pompidou Hospital, AP-HP Centre, University of Paris, Paris, France; 11 Medical Oncology, Corporación Sanitaria Parc Tauli, Barcelona, Spain; 12 Department of Internal Medicine, Division of Medical Oncology, Clinical Trial Unit, Cerrahpaşa School of Medicine, Istanbul University-Cerrahpaşa, Istanbul, Turkey; 13 Medical Oncology Department, Vall d’Hebron Institute of Oncology (VHIO), Barcelona, Spain; 14 Stathmi, Inc., New Hope, PA USA; 15 Clinical Development, Taiho Oncology, Inc., Princeton, NJ USA; 16 Gastrointestinal Oncology Service in the Department of Medicine, Memorial Sloan Kettering Cancer Center, New York, NY USA

**Keywords:** Stomach neoplasms, Gastrointestinal neoplasms, Trifluridine tipiracil, Age groups, Aged, Randomized controlled trial

## Abstract

**Background:**

Trifluridine and tipiracil (FTD/TPI) demonstrated survival benefit vs placebo and manageable safety in previously treated patients with metastatic gastric/gastroesophageal junction cancer (mGC/GEJC) in the randomized, placebo-controlled, phase 3 TAGS study. This subgroup analysis of TAGS examined efficacy/safety outcomes by age.

**Methods:**

In TAGS, patients with mGC/GEJC and ≥ 2 prior therapies were randomized (2:1) to receive FTD/TPI 35 mg/m^2^ or placebo, plus best supportive care. A preplanned subgroup analysis was performed to evaluate efficacy and safety outcomes in patients aged < 65, ≥ 65, and ≥ 75 years.

**Results:**

Among 507 randomized patients (*n* = 337 FTD/TPI; *n* = 170 placebo), 55%, 45%, and 14% were aged < 65, ≥ 65, and ≥ 75 years, respectively. Overall survival hazard ratios for FTD/TPI vs placebo were 0.67 (95% CI 0.51–0.89), 0.73 (95% CI 0.52–1.02), and 0.67 (95% CI 0.33–1.37) in patients aged < 65, ≥ 65, and ≥ 75 years, respectively. Regardless of age, patients receiving FTD/TPI experienced improved progression-free survival and stayed longer on treatment than those receiving placebo. Among FTD/TPI-treated patients, frequencies of any-cause grade ≥ 3 adverse events (AEs) were similar across age subgroups (80% each), although grade ≥ 3 neutropenia was more frequent in older patients [40% (≥ 65 and ≥ 75 years); 29% (< 65 years)]; AE-related discontinuation rates did not increase with age [14% (< 65 years), 12% (≥ 65 years), and 12% (≥ 75 years)].

**Conclusions:**

The results of this subgroup analysis show the efficacy and tolerability of FTD/TPI treatment regardless of age in patients with mGC/GEJC who had received 2 or more prior treatments.

**Supplementary Information:**

The online version contains supplementary material available at 10.1007/s10120-021-01271-9.

## Introduction

Gastric cancer is the fourth leading cause of cancer-related deaths worldwide, with a million new cases and 769,000 deaths reported in 2020 [1]. Gastric cancer is often considered a disease of the elderly, as nearly 60% of newly diagnosed patients with gastric cancer are ≥ 65 years, and about one-third are ≥ 75 years [2, 3]. Older patients have a higher incidence of comorbidities and are at a higher risk of adverse outcomes after surgery than younger patients [4, 5]. Therefore, they are often less likely to receive recommended treatment and suffer higher mortality rates [5, 6]. As these patients remain largely underrepresented in gastric cancer trials, there is a paucity of prospective data on chemotherapy in this patient population, particularly in later line settings [3, 7].

Trifluridine and tipiracil (FTD/TPI), an oral cytotoxic chemotherapy indicated for previously treated metastatic colorectal cancer [8, 9], received approval in 2019 (in US, Japan, and Europe) for the treatment of patients with previously treated metastatic gastric/gastroesophageal junction adenocarcinoma (mGC/GEJC) [10]. This approval was based on results from the phase 3 TAGS study [11] that demonstrated a significant survival benefit from FTD/TPI treatment versus placebo [median overall survival (OS) 5.7 vs 3.6 months; hazard ratio (HR), 0.69; *P* = 0.00058] in patients with mGC/GEJC who had received ≥ 2 prior systemic therapies. The most common adverse events (AEs) with FTD/TPI treatment were hematologic in nature (neutropenia and anemia) and FTD/TPI had a manageable safety profile: most AEs were managed with dosing adjustments, and discontinuations due to AEs occurred in only 12% of patients. Analysis of patient-reported outcomes showed that quality of life (QoL) was maintained in the FTD/TPI arm of the TAGS study, and a trend toward reduced risk of deterioration in QoL scores was noted with FTD/TPI treatment compared with placebo [12].

In the overall population of the phase 3 TAGS study, the median age was 63.0 years, with 45% of patients aged ≥ 65 years [11]. In this subgroup analysis of the TAGS study, we examined efficacy and safety outcomes by age (< 65, ≥ 65, and ≥ 75 years).

## Materials and methods

### Study design

In this prespecified subgroup analysis of the TAGS study, a global randomized double-blind placebo-controlled phase 3 study that was conducted in 17 countries at 110 sites between February 24, 2016, and January 5, 2018 [11], the efficacy of FTD/TPI versus placebo was evaluated according to age (< 65, ≥ 65, and ≥ 75 years) in previously treated patients with mGC/GEJC. The TAGS study was conducted in accordance with the Declaration of Helsinki and Good Clinical Practice Guidelines as specified by the International Conference on Harmonisation. The protocol was approved by the institutional review boards or independent ethics committees at each participating center, and all patients provided written informed consent.

### Patients and treatment

Eligible patients were aged ≥ 18 years, with histologically confirmed nonresectable metastatic gastric adenocarcinoma or adenocarcinoma of the gastroesophageal junction, had received at least 2 prior treatment regimens for advanced disease, and were refractory or intolerant to their most recent therapy. Patients also had to have an Eastern Cooperative Oncology Group (ECOG) performance status (PS) of 0 or 1. Additional exclusion/inclusion criteria have been described previously [11].

Patients were randomized in a 2:1 ratio to receive either FTD/TPI 35 mg/m^2^ administered orally on days 1–5 and 8–12 of a 28-day cycle plus best supportive care or placebo plus best supportive care. Treatment was continued until disease progression, unacceptable toxicity, or patient withdrawal.

### Endpoints

The primary endpoint of the phase 3 TAGS study was OS. Secondary endpoints included progression-free survival (PFS), safety, and tolerability. Other endpoints included time to deterioration of ECOG PS (time from randomization until an ECOG PS score of 2 or higher was recorded), objective response rate, disease control rate, and health-related QoL. In this subgroup analysis, time to treatment discontinuation (due to any cause) was also assessed.

### Assessments

Tumor response was assessed by the investigator per the revised Response Evaluation Criteria in Solid Tumors version 1.1. Tumor assessments were performed within 28 days prior to day 1 of cycle 1 and every 8 weeks thereafter until patient discontinuation due to disease progression. Patients who discontinued for other reasons were followed for tumor response every 8 weeks. Patients were assessed for safety from the time of signed consent until 30 days after last dose of study treatment. AEs were graded according to National Cancer Institute Common Terminology Criteria for Adverse Events (NCI-CTCAE) version 4.03.

### Statistical considerations

Statistical considerations for the overall phase 3 TAGS study have been described previously [11]. Briefly, the study was designed to detect an HR for death of 0.70 for FTD/TPI vs placebo with a 90% power and a 1-sided type 1 error rate of 0.025. A total of 384 deaths were targeted for the final OS analysis.

All three age subgroups (< 65, ≥ 65, and ≥ 75 years) were prespecified in the study protocol or statistical analysis plan, and age (< 65 years vs ≥ 65 years) was included as a prespecified factor in the multivariate analysis of OS (the primary endpoint). All patients included in the intent-to-treat assessment of the TAGS study were included in the efficacy analysis. All patients who received ≥ 1 study drug dose were included in the safety analysis. Although planned, these subgroup analyses were not powered for statistical significance, and no formal comparisons were made between the age subgroups. For time to event endpoints (OS, PFS, time to deterioration of ECOG PS, or time to discontinuation of treatment), Kaplan–Meier estimates of the medians and specific time points and HRs with their corresponding 95% confidence intervals (CIs) calculated using a Cox proportional hazard model were provided, but no *P* values were included. All statistical analyses were conducted using SAS statistical software, version 9.4.

## Results

### Patient population and disposition

Of 507 patients who were enrolled and randomized (337 to FTD/TPI and 170 to placebo), 279 patients (55%) were aged < 65 years, 228 (45%) were aged ≥ 65 years, and 69 (14%) were aged ≥ 75 years (Table 1). Patient baseline characteristics were generally similar across the younger and older age subgroups, although a few key differences were noticed. A greater proportion of patients in the older age subgroups than in the younger subgroup had moderate renal impairment (41%, 31%, and 6% in the ≥ 75-, ≥ 65-, and < 65-year subgroups, respectively). Second, a greater proportion of patients in the older age subgroups (66% and 74% in the ≥ 65-year and ≥ 75-year subgroups, respectively) than in the < 65-year subgroup (59%) had an ECOG PS of 1. Also, a somewhat higher proportion of patients in the ≥ 65-year and ≥ 75-year subgroups (29% and 28%) had received ≥ 4 prior regimens than in the < 65-year subgroup (21%). Baseline characteristics were comparable between treatment arms for all three subgroups, except for the following imbalance in ECOG PS within the ≥ 65-year subgroup: 69% of patients in the FTD/TPI arm had an ECOG PS of 1 versus 59% in the placebo arm.

**Table 1:** Baseline patient and disease characteristics^a^

	< 65 years		≥ 65 years		≥ 75 years	
	FTD/TPI (*n* = 183)	Placebo (*n* = 96)	FTD/TPI (*n* = 154)	Placebo (*n* = 74)	FTD/TPI (*n* = 51)	Placebo (*n* = 18)
Age, years						
Mean (SD)	55.1 (7.9)	55.2 (7.5)	71.9 (5.1)	70.9 (4.4)	78.1 (3.0)	77.0 (2.1)
Median (range)	57 (24‒64)	57 (32‒64)	71.0 (65‒89)	70.0 (65‒82)	78.0 (75–89)	76.0 (75–82)
Sex, *n* (%)						
Male	140 (77)	69 (72)	112 (73)	48 (65)	36 (71)	10 (56)
Female	43 (23)	27 (28)	42 (27)	26 (35)	15 (29)	8 (44)
Race, *n* (%)						
White	139 (76)	65 (68)	105 (68)	48 (65)	36 (71)	15 (83)
Asian	20 (11)	13 (14)	31 (20)	16 (22)	9 (18)	1 (6)
Other	3 (2)	3 (3)	1 (< 1)	1 (1)	0	0
Not collected	21 (11)	15 (16)	17 (11)	9 (12)	6 (12)	2 (11)
Geographic region, *n* (%)						
Europe	156 (85)	82 (85)	114 (74)	56 (76)	38 (75)	16 (89)
Japan	15 (8)	11 (11)	31 (20)	16 (22)	9 (18)	1 (6)
USA	12 (7)	3 (3)	9 (6)	2 (3)	4 (8)	1 (6)
ECOG PS, *n* (%)						
0	76 (42)	38 (40)	47 (31)	30 (41)	14 (27)	4 (22)
1	107 (58)	58 (60)	107 (69)	44 (59)	37 (73)	14 (78)
Renal function, *n* (%)						
Normal (≥ 90 mL/min)	103 (56)	49 (51)	31 (20)	19 (26)	5 (10)	1 (6)
Mild impairment (60–89 mL/min)	70 (38)	40 (42)	71 (46)	31 (42)	22 (43)	10 (56)
Moderate impairment (30–59 mL/min)	9 (5)	7 (7)	49 (32)	21 (28)	22 (43)	6 (33)
Severe impairment (< 30 mL/min)	0	0	2 (1)	1 (1)	1 (2)	0
Missing	1 (< 1)	0	1 (< 1)	2 (3)	1 (2)	1 (6)
Hepatic function (NCI-ODWG criteria), *n* (%)						
Normal	136 (74)	74 (77)	112 (73)	57 (77)	40 (78)	13 (72)
Mild impairment	44 (24)	17 (18)	40 (26)	15 (20)	11 (22)	5 (28)
Moderate impairment	1 (< 1)	1 (1)	1 (< 1)	0	0	0
Severe impairment	0	1 (1)	0	0	0	0
Missing	2 (1)	3 (3)	1 (< 1)	2 (3)	0	0
Number of metastatic sites, *n* (%)						
≤ 2	73 (40)	39 (41)	82 (53)	33 (45)	32 (63)	12 (67)
≥ 3	110 (60)	57 (59)	72 (47)	41 (55)	19 (37)	6 (33)
Prior gastrectomy, *n* (%)	74 (40)	43 (45)	73 (47)	31 (42)	20 (39)	6 (33)
Number of prior regimens,^b^ *n* (%)						
2	76 (42)	41 (43)	50 (32)	23 (31)	18 (35)	7 (39)
3	72 (39)	32 (33)	62 (40)	28 (38)	19 (37)	6 (33)
≥ 4	35 (19)	23 (24)	42 (27)	23 (31)	14 (27)	5 (28)
Prior systemic anticancer agents,^b^ *n* (%)						
Fluoropyrimidine	183 (100)	96 (100)	153 (99)	74 (100)	50 (98)	18 (100)
Platinum	183 (100)	96 (100)	154 (100)	74 (100)	51 (100)	18 (100)
Irinotecan	96 (52)	51 (53)	87 (56)	47 (64)	28 (55)	10 (56)
Taxane	168 (92)	82 (85)	143 (93)	66 (89)	45 (88)	17 (94)
Ramucirumab	54 (30)	24 (25)	60 (39)	31 (42)	15 (29)	5 (28)

*ECOG PS* Eastern Cooperative Oncology Group performance status, *FTD/TPI* trifluridine/tipiracil, *NCI-ODWG* National Cancer Institute organ dysfunction working group, *SD* standard deviation

^a^Intent-to-treat population

^b^In any setting (neoadjuvant, adjuvant, or metastatic)

Patient disposition was similar across the < 65-, ≥ 65-, and ≥ 75-year subgroups: at data cutoff (March 31, 2018), 96%, 93%, and 90% of FTD/TPI-treated patients in the respective subgroups discontinued treatment (Supplementary Table S1). The most common reason for discontinuation was disease progression across all subgroups (ranging from 72% to 76% among FTD/TPI-treated patients).

### Time to discontinuation and treatment exposure

Mean FTD/TPI dose intensities were similar across the < 65-, ≥ 65-, and ≥ 75-year subgroups (146.1, 150.7, and 149.3 mg/m^2^/week, respectively), but median treatment duration was observed to be somewhat longer among older patients (6.0, 7.6, and 9.6 weeks, respectively), and cumulative FTD/TPI doses were marginally higher in the ≥ 65- and ≥ 75-year subgroups than in the < 65-year subgroup (Supplementary Table S2).

**Fig. 1:**
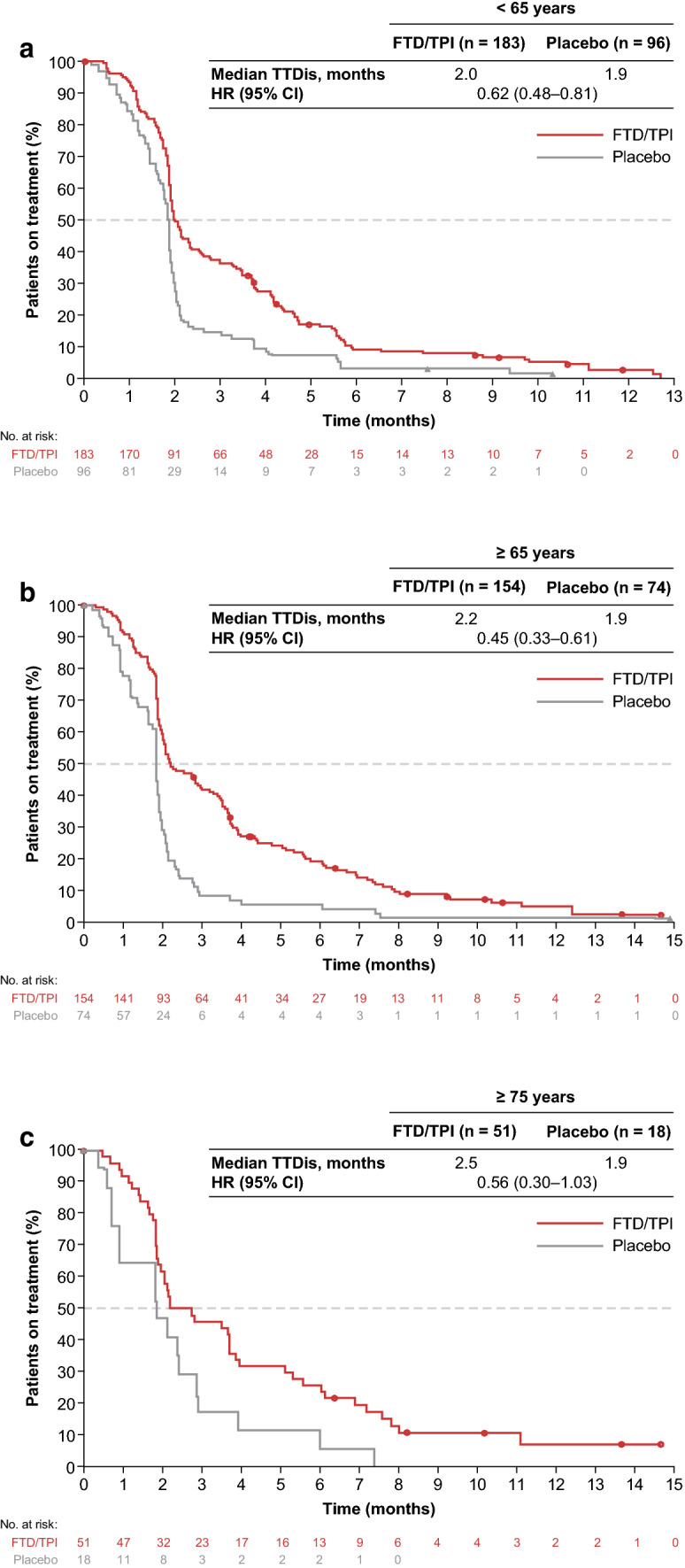
Time to treatment discontinuation due to any cause in patients aged **a** < 65 years, **b** ≥ 65 years, and **c** ≥ 75 years. *FTD/TPI* trifluridine/tipiracil, *HR* hazard ratio, *TTDis* time to treatment discontinuation

Across all 3 age subgroups, patients receiving FTD/TPI stayed longer on treatment than those receiving placebo. Median time to treatment discontinuation in the FTD/TPI versus placebo groups among patients aged < 65 years was 2.0 vs 1.9 months with an HR of 0.62 (95% CI 0.48–0.81). In patients aged ≥ 65 years and ≥ 75 years, the corresponding values were 2.2 vs 1.9 months with a HR of 0.45 (95% CI 0.33–0.61), and 2.5 vs 1.9 months with a HR of 0.56 (95% CI 0.30–1.03; Fig. 1). These results were consistent with that observed in the overall population (2.1 vs 1.9 months; HR 0.54; 95% CI 0.44–0.66).

### Efficacy

As previously reported, in the overall patient population, FTD/TPI treatment significantly improved both OS and PFS in patients with mGC/GEJC compared with placebo [11]. In multivariate Cox regression analyses of OS that included region, ECOG PS at baseline, and prior ramucirumab treatment as stratification factors, age (< 65 years vs ≥ 65 years) was identified as a prognostic factor (*P* = 0.0003) but was not predictive of OS (*P*_*interactio*n_ = 0.55).

**Fig. 2:**
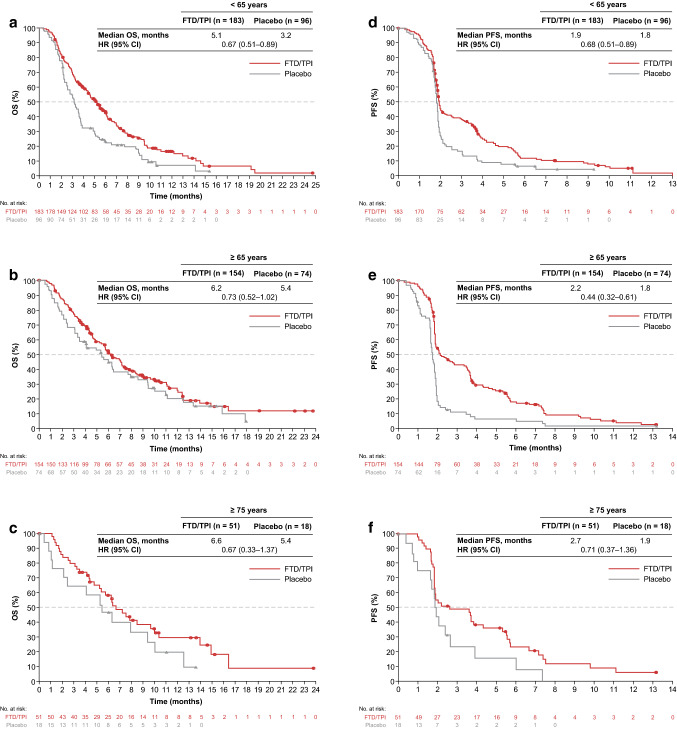
Overall survival (**a**, **b**, **c**) and progression-free survival (**d**, **e**, **f**) in patients aged < 65, ≥ 65, and ≥ 75 years, respectively. *FTD/TPI* trifluridine/tipiracil, *HR* hazard ratio, *OS* overall survival, *PFS* progression-free survival

In the younger subgroup (aged < 65 years), FTD/TPI treatment showed OS benefit vs placebo, similar to observations in the overall population; median OS was 5.1 vs 3.2 months in the FTD/TPI vs placebo groups, with an HR of 0.67 (95% CI 0.51–0.89; Fig. 2a). OS HRs also favored FTD/TPI vs placebo in patients aged ≥ 65 years [0.73 (95% CI 0.52–1.02); median OS, 6.2 vs 5.4 months], and those aged ≥ 75 years [0.67 (95% CI 0.33–1.37); median OS, 6.6 vs 5.4 months], although the OS HR confidence intervals were somewhat wider in the ≥ 75-year subgroup (Fig. 2b and c).

Similarly, PFS HRs favored FTD/TPI over placebo in all age subgroups. In the < 65-year and ≥ 65-year subgroups, PFS HRs with FTD/TPI vs placebo were 0.68 (95% CI 0.51–0.89) and 0.44 (95% CI 0.32–0.61), indicating PFS benefits in both subgroups. In the ≥ 75-year subgroup, the PFS HR was 0.71 (95% CI 0.37–1.36) (Fig. 2d–f).

Because renal impairment was a potential confounding factor in older patients (75% of patients aged ≥ 65 years had mild-to-moderate renal impairment compared with 45% of patients aged < 65 years), exploratory post hoc analyses were carried out to examine the effect of renal impairment on OS and PFS in older patients (Supplementary Table S3). FTD/TPI treatment showed PFS benefit versus placebo in older patients regardless of renal impairment: PFS HRs were 0.31 (95% CI 0.14–0.66), 0.49 (0.29–0.81), and 0.60 (0.33–1.10) in patients with normal renal function, mild renal impairment, and moderate renal impairment, respectively. OS benefit with FTD/TPI versus placebo was apparent in older patients with normal renal function [median OS, 5.7 vs 3.9 months; HR 0.71 (95% CI 0.33–1.57)] and in patients with moderate renal impairment [median OS, 6.3 vs 4.1 months; HR 0.79 (0.42–1.48)], but appeared marginal in patients with mild renal impairment [median OS, 6.2 vs 6.3 months; HR 0.86 (0.50–1.49)]. This could likely be attributed to an imbalance in ECOG PS favoring the placebo arm among older patients with mild renal impairment: 52% and 48% of patients in the placebo group had an ECOG PS of 0 and 1, respectively, compared with 30% and 70% in the FTD/TPI group.

**Fig. 3:**
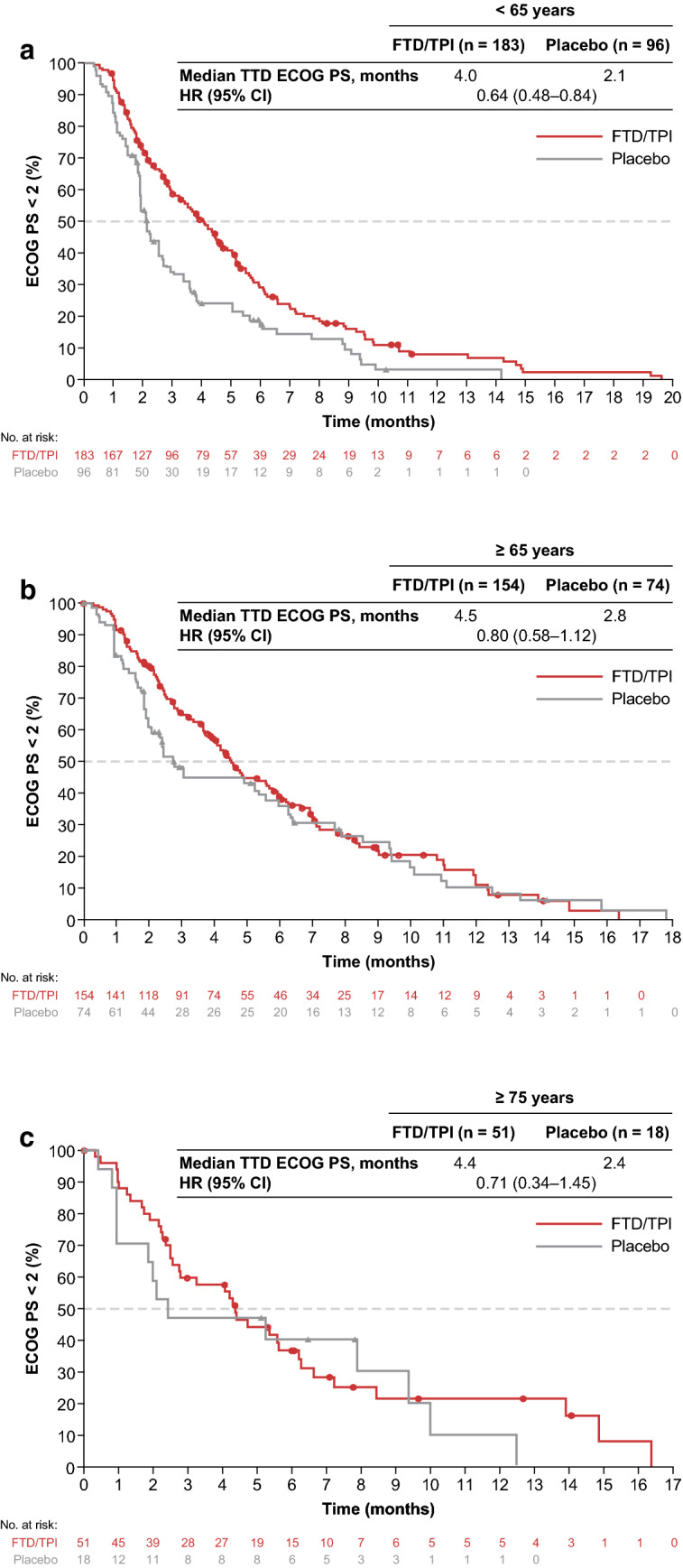
Time to deterioration of ECOG PS to 2 or higher in patients aged **a** < 65 years, **b** ≥ 65 years, and **c** ≥ 75 years. *ECOG PS* Eastern Cooperative Oncology Group performance status, *FTD/TPI* trifluridine/tipiracil, *HR* hazard ratio, *TTD* time to deterioration

ECOG PS was maintained longer with FTD/TPI treatment than with placebo in the TAGS study. Although this difference was most pronounced in the younger (< 65-year) subgroup, where the median time to deterioration to an ECOG PS of ≥ 2 was 4.0 vs 2.1 months for FTD/TPI versus placebo (HR 0.64; 95% CI 0.48–0.84; Fig. 3a), similar trends were observed in the older subgroups. In the ≥ 65-year and ≥ 75-year subgroups, respectively, HRs for time to deterioration of ECOG PS in the FTD/TPI vs placebo groups were 0.80 (95% CI 0.58–1.12; median, 4.5 vs 2.8 months) and 0.71 (95% CI 0.34–1.45; median, 4.4 vs 2.4 months; Fig. 3b and c).

### Safety

The overall incidences of AEs and grade ≥ 3 AEs with FTD/TPI treatment (80% in each subgroup) were similar in younger and older patients (Table 2; Supplementary Table S4). The most common AEs with FTD/TPI treatment were neutropenia (occurring in 49%, 56%, and 56% of patients in the < 65-, ≥ 65-, and ≥ 75-year subgroups, respectively), nausea (46%, 26%, and 26%), anemia (44%, 46%, and 54%), and decreased appetite (32%, 37%, and 42%; Supplementary Fig. S1). The occurrence of hematologic toxicities, including neutropenia, anemia, leukopenia, and thrombocytopenia, was specific to FTD/TPI treatment, and rarely occurred with placebo as shown in the odds ratio plots in Supplementary Fig. S2. This treatment effect was consistently observed across all age subgroups.

**Table 2:** Safety summary and adverse events of any cause in ≥ 10% of patients in any group^a^

	Number of patients (%)					
	< 65 years		≥ 65 years		≥ 75 years	
	FTD/TPI (*n* = 182)	Placebo (*n* = 96)	FTD/TPI (*n* = 153)	Placebo (*n* = 72)	FTD/TPI (*n* = 50)	Placebo (*n* = 17)
AEs of any cause	179 (98)	91 (95)	147 (96)	66 (92)	50 (100)	17 (100)
Grade ≥ 3 AEs	145 (80)	60 (62)	122 (80)	37 (51)	40 (80)	9 (53)
Treatment-related AEs	149 (82)	59 (61)	122 (80)	36 (50)	40 (80)	9 (53)
Grade ≥ 3 treatment-related AEs	89 (49)	14 (15)	87 (57)	8 (11)	28 (56)	2 (12)
Actions taken because of any-grade AEs of any cause						
Dosing modification	101 (55)	21 (22)	94 (61)	16 (22)	32 (64)	3 (18)
Discontinuation	25 (14)	18 (19)	18 (12)	10 (14)	6 (12)	4 (24)
AEs of any cause in ≥ 10% of patients in any group						
Hematologic						
Neutropenia^b^	90 (49)	4 (4)	86 (56)	3 (4)	28 (56)	0
Anemia^c^	80 (44)	21 (22)	70 (46)	11 (15)	27 (54)	4 (24)
Leukopenia^d^	43 (24)	2 (2)	35 (23)	1 (1)	16 (32)	0
Thrombocytopenia^e^	30 (16)	4 (4)	30 (20)	4 (6)	10 (20)	1 (6)
Gastrointestinal						
Nausea	84 (46)	38 (40)	40 (26)	15 (21)	13 (26)	3 (18)
Vomiting	53 (29)	20 (21)	30 (20)	14 (19)	10 (20)	3 (18)
Diarrhea	40 (22)	13 (14)	36 (24)	11 (15)	11 (22)	1 (6)
Abdominal pain	31 (17)	19 (20)	24 (16)	12 (17)	7 (14)	3 (18)
Constipation	24 (13)	17 (18)	21 (14)	8 (11)	8 (16)	3 (18)
Upper abdominal pain	16 (9)	10 (10)	6 (4)	5 (7)	2 (4)	0
Ascites	12 (7)	12 (12)	7 (5)	4 (6)	2 (4)	1 (6)
Dysphagia	12 (7)	4 (4)	8 (5)	4 (6)	2 (4)	2 (12)
Gastric hemorrhage	3 (2)	1 (1)	0	3 (4)	0	2 (12)
Other AEs						
Decreased appetite	58 (32)	35 (36)	57 (37)	17 (24)	21 (42)	3 (18)
Fatigue	50 (27)	19 (20)	39 (25)	16 (22)	13 (26)	7 (41)
Asthenia	31 (17)	23 (24)	34 (22)	17 (24)	11 (22)	3 (18)
Increased blood alkaline phosphatase	16 (9)	5 (5)	14 (9)	9 (12)	5 (10)	2 (12)
Pyrexia	16 (9)	4 (4)	9 (6)	4 (6)	5 (10)	1 (6)
Dyspnea	15 (8)	9 (9)	9 (6)	8 (11)	3 (6)	2 (12)
General physical health deterioration	15 (8)	10 (10)	8 (5)	7 (10)	2 (4)	1 (6)
Decreased weight	10 (5)	9 (9)	10 (7)	3 (4)	5 (10)	0
Hyperglycemia	7 (4)	2 (2)	2 (1)	3 (4)	0	2 (12)
Peripheral edema	7 (4)	6 (6)	10 (7)	6 (8)	2 (4)	2 (12)
Cough	6 (3)	3 (3)	5 (3)	3 (4)	2 (4)	2 (12)
Urinary tract infection	3 (2)	1 (1)	6 (4)	4 (6)	5 (10)	0

*AE* adverse event; *FTD/TPI* trifluridine/tipiracil

^a^As-treated population

^b^Includes decreased neutrophil count

^c^Includes decreased hemoglobin

^d^Includes decreased white blood cell count

^e^Includes decreased platelet count

Although AE incidences with FTD/TPI treatment were largely similar across older and younger patients (Table 2, Supplementary Fig. S1), a few differences were noted: hematologic toxicities (neutropenia and anemia) tended to be more frequent in older patients than in younger patients (Supplementary Fig. S1). Grade ≥ 3 neutropenia occurred in 40% of patients each in the ≥ 65- and ≥ 75-year subgroups and in 29% of patients in the < 65-year subgroup. Decreased appetite (anorexia) showed a slight trend of increase with age (32%, 37%, and 42% in patients aged < 65, ≥ 65, and ≥ 75 years, respectively). Nausea (of any grade) was more frequent in the < 65-year subgroup (46% of patients) than in the ≥ 65- and ≥ 75-year subgroups (26% each). AEs were managed well with dosing adjustments and supportive medications in both younger and older patients. Overall, 55%, 61%, and 64% of patients in the < 65-, ≥ 65-, and ≥ 75-year subgroups had dosing modifications (dosing delays or dose reductions) due to AEs of any cause (Table 2). Higher proportions of older patients received supportive medications for neutropenia (15%, 20%, and 28% in the < 65-, ≥ 65-, and ≥ 75-year subgroups, respectively) and anemia (17%, 20%, and 28%, respectively) than younger patients. AE-related discontinuation rates did not increase with age: among FTD/TPI-treated patients, treatment discontinuations due to AEs of any cause were reported in 14% of patients aged < 65 years and in 12% each of patients aged ≥ 65 and ≥ 75 years.

An exploratory post hoc analysis of safety in older patients (aged ≥ 65 years) by renal function indicated that overall rates of AEs and grade ≥ 3 AEs were similar in patients with normal renal function or mild-to-moderate renal impairment (Supplementary Table S5), although certain hematologic AEs (neutropenia, anemia, and thrombocytopenia) were more frequent in FTD/TPI-treated patients with mild or moderate renal impairment than in those with normal renal function. Despite these variations in the AE profile, the overall rates of dosing modifications were similar across the renal function subgroups (59% to 63%) and AE-related drug discontinuations did not increase as renal function worsened (13%, 8%, and 16% in patients with normal renal function, mild renal impairment, and moderate renal impairment, respectively).

## Deterioration in global health status scores by age

The median time to deterioration by ≥ 5 points in the European Organization for the Research and Treatment of Cancer Quality of Life (EORTC-QLQ-C30) global health scores with FTD/TPI is shown in Supplementary Table S6. For the < 65-, ≥ 65-, and ≥ 75-year subgroups, respectively, the time to deterioration HRs for FTD/TPI versus placebo were 1.30 (95% CI 0.70–2.43), 1.64 (0.80–3.36), and 0.78 (0.07–8.88), suggesting that the changes in global health status scores were not markedly different between FTD/TPI and placebo regardless of age.

## Discussion

The results of this subgroup analysis indicated that FTD/TPI treatment resulted in efficacy benefits in patients with mGC/GEJC in the TAGS study regardless of age. Improvements in OS and PFS were observed in both younger (< 65-year-old) and older (≥ 65- and ≥ 75-year-old) patients with FTD/TPI compared with placebo. Although the OS benefit appeared to be somewhat less pronounced in the ≥ 75-year subgroup, the smaller patient numbers in this subgroup limited the interpretation of this result. ECOG PS was maintained longer with FTD/TPI than with placebo across all age subgroups, even though the difference between the FTD/TPI and placebo groups was more pronounced in younger patients. As deterioration in ECOG PS was significantly associated with deterioration in QoL scores in the TAGS study [12], it may be reasonably assumed that a trend toward slower deterioration in QoL with FTD/TPI treatment was observed irrespective of age. Consistent with the efficacy benefits observed, patients of all age subgroups who were randomized to FTD/TPI stayed longer on treatment than those randomized to placebo. Overall, these data are consistent with the previous reports in metastatic colorectal cancer, which showed similar efficacy benefits with FTD/TPI treatment in younger and older (≥ 65-year-old) patients [13, 14].

The safety of FTD/TPI treatment did not appear to be impacted by patient age in the TAGS study. The FTD/TPI safety profile was consistent across younger and older age subgroups, with similar overall grade ≥ 3 AE incidences and no unexpected safety concerns. A trend of increase in frequency of decreased appetite (anorexia) was noted with increasing age, indicating that anorexia may require careful management in elderly patients. Hematologic toxicities, including neutropenia and anemia, increased in frequency with age. It is likely that increasing comorbidities among older patients, including renal impairment, may have accounted for this increasing trend: more FTD/TPI-treated patients in the ≥ 65- and ≥ 75-year subgroups had moderate-to-severe renal impairment (33% and 45%) compared with the < 65-year subgroup (5%). This was confirmed in the additional analyses of safety by renal impairment in older patients (aged ≥ 65 years). The incidences of neutropenia and anemia were higher in patients with mild or moderate renal impairment than in patients with normal renal function. In both younger and older patients, however, toxicities were managed with dosing adjustments and/or supportive medications. Treatment discontinuation rates related to AEs were not higher in the older subgroups than in younger patients. Renal impairment did not appear to affect dosing modification or treatment discontinuation rates among older patients. In an analysis of patient quality of life by age, no specific trend emerged.

Patients aged ≥ 65 years constitute the majority of the gastric cancer patient population in the real world [2, 3, 6]. The presence of comorbidities and the increased risk of toxicities [4, 5] have often deterred physicians from pursuing aggressive treatment options, including systemic chemotherapy, in older patients [3, 6, 7]. Yet, increasing evidence suggests that chemotherapy and other systemic therapies may be well tolerated in older patients [3, 15–18]. Multiple studies have reported the efficacy and tolerability of first-line combination chemotherapy regimens in patients with advanced or metastatic gastric cancer who were aged ≥ 65 years [15–17]. More recently, results from a subgroup analysis of the phase 3 RAINBOW and REGARD trials of second-line ramucirumab in patients with mGC/GEJC suggested that age did not influence the efficacy or safety of ramucirumab in these patients [18]. Although limited data on third- or later-line therapy in older patients are suggestive of efficacy with systemic anticancer therapy in these patients, data on safety remain lacking [19, 20]. The current analysis in the TAGS study is among the most detailed for third- or later-line therapy in this patient population and demonstrates both efficacy and safety of FTD/TPI in heavily pretreated older patients. Renal impairment did not appear to have a major impact on efficacy or safety outcomes with FTD/TPI in older patients, although these analyses were limited by their exploratory post hoc nature and small patient numbers. Together, these data reiterate the point that systemic therapies should be actively considered in older patients, including for treatment beyond second line.

One of the main limitations of this analysis was that it was not powered for statistical significance, even though the subgroups were prespecified. As a result, the analysis did not include a robust comparison of efficacy and safety between younger and older patients. In addition, the older subpopulation in the TAGS study, which constituted 45% of the overall patient population (with patients aged ≥ 75 years constituting ~ 14%), cannot be considered strictly representative of the older mGC/GEJC population. According to the Surveillance, Epidemiology, and End Results (SEER) database, ~ 60% of cases develop in patients aged ≥ 65 years, with ~ 33% in patients aged ≥ 75 years [21]. In addition, in real-world clinical practice, many patients would have been unlikely to fulfill the inclusion criteria (ECOG PS of 0 or 1, adequate organ function) specified in the TAGS study. A comprehensive geriatric assessment was not utilized in the study, [22], so it is not clear how the older subpopulation compared with older patients in routine clinical practice. Real-world studies, similar to those performed in colorectal cancer [14], will be helpful to assess the safety and efficacy of FTD/TPI among older patients with gastric cancer.

In conclusion, the results of this detailed subgroup analysis show the efficacy and tolerability of FTD/TPI treatment regardless of age in patients with mGC/GEJC who had received 2 or more prior treatments. FTD/TPI can be considered a safe and effective treatment option in heavily pretreated patients with mGC/GEJC aged ≥ 65 years.

## Supplementary Information

Below is the link to the electronic supplementary material.

Supplementary file1 (DOCX 283 KB)
