# Supplementary material for: Efficacy and safety of trifluridine/tipiracil in older and younger patients with metastatic gastric or gastroesophageal junction cancer: subgroup analysis of a randomized phase 3 study (TAGS)
Source: Gastric Cancer. 2022 Jan 8;25(3):586–97. doi: 10.1007/s10120-021-01271-9 (PMC9013328; doi:10.1007/s10120-021-01271-9)

Efficacy and safety of trifluridine/tipiracil in older and younger patients with metastatic gastric or gastroesophageal junction cancer: subgroup analysis of a randomized phase 3 study (TAGS)

Kohei Shitara,Toshihiko Doi, Hisashi Hosaka, Peter Thuss-Patience, Armando Santoro, Federico Longo, Ozgur Ozyilkan, Irfan Cicin, David Park, Aziz Zaanan, Carles Pericay, Mustafa Özgüroğlu, Maria Alsina, Lukas Makris, Karim A. Benhadji, David H. Ilson

**Corresponding author**: Kohei Shitara

Department of Gastrointestinal Oncology, National Cancer Center Hospital East

[kshitara@east.ncc.go.jp](mailto:kshitara@east.ncc.go.jp)

## **Journal Name**: Gastric Cancer

# Supplementary Data

# Table S1 Patient disposition

|  | **Number of patients (%)** | | | | | |
| --- | --- | --- | --- | --- | --- | --- |
|  | **< 65 years** | | **≥ 65 years** | | **≥ 75 years** | |
|  | **FTD/TPI** | **Placebo** | **FTD/TPI** | **Placebo** | **FTD/TPI** | **Placebo** |
| **Intent-to-treat population** | 183 (100) | 96 (100) | 154 (100) | 74 (100) | 51 (100) | 18 (100) |
| **As-treated population** | 182 (99) | 96 (100) | 153 (99) | 72 (97) | 50 (98) | 17 (94) |
| Ongoing^a^ | 8 (4) | 2 (2) | 11 (7) | 1 (1) | 5 (10) | 0 |
| Discontinued study treatment^a^ | 174 (96) | 94 (98) | 142 (93) | 71 (99) | 45 (90) | 17 (100) |
| Radiological/clinical progression^a^ | 131 (72) | 80 (83) | 115 (75) | 65 (90) | 38 (76) | 15 (88) |
| Adverse event^a^ | 22 (12) | 8 (8) | 11 (7) | 3 (4) | 4 (8) | 1 (6) |
| Patient withdrew consent^a^ | 9 (5) | 3 (3) | 5 (3) | 1 (1) | 1 (2) | 1 (6) |
| Physician’s decision^a^ | 4 (2) | 2 (2) | 7 (5) | 1 (1) | 2 (4) | 0 |
| Death^a^ | 8 (4) | 1 (1) | 3 (2) | 1 (1) | 0 | 0 |
| Other^a^ | 0 | 0 | 1 (<1) | 0 | 0 | 0 |

*FTD/TPI* trifluridine/tipiracil.

^a^Percentage of the as-treated population.

# Table S2 Study drug exposure^a^

|  |  | | | | | |
| --- | --- | --- | --- | --- | --- | --- |
|  | **< 65 years** | | **≥ 65 years** | | **≥ 75 years** | |
|  | **FTD/TPI**  **(n = 182)** | **Placebo**  **(n = 96)** | **FTD/TPI**  **(n = 153)** | **Placebo (n = 72)** | **FTD/TPI**  **(n = 50)** | **Placebo**  **(n = 17)** |
| Cycles initiated, median (range) | 2 (1–12) | 2 (1–12) | 2 (1–14) | 2 (1–16) | 3 (1–14) | 2 (1–8) |
| Median duration of treatment (range), weeks | 6.0 (0.4–52.4) | 5.7 (0.1–46.0) | 7.6 (0.7–62.7) | 5.7 (0.7–63.0) | 9.6 (1.1–62.7) | 5.7 (1.3–29.7) |
| Median relative dose intensity (range) | 0.90 (0.15–1.01) | 0.96 (0.10–1.04) | 0.89 (0.43–1.01) | 0.94 (0.42–1.10) | 0.87 (0.43–1.01) | 0.94 (0.42–1.02) |
| Mean (SD) dose intensity, mg/m^2^/wk | 146.1 (30.6) | 155.3 (30.1) | 150.7 (21.2) | 154.5 (24.8) | 149.3 (21.2) | 153.8 (27.3) |
| Mean cumulative dose (SD), mg/m^2^ | 1995.9 (1575.0) | 1505.9 (1239.7) | 2284.6 (1731.2) | 1520.2 (1440.5) | 2627.0 (2057.7) | 1678.1 (1347.0) |

*FTD/TPI* trifluridine/tipiracil; *SD* standard deviation; *wk* week.

^a^As-treated population.

**Table S3** OS and PFS by renal function status in patients aged ≥ 65 years^a^

|  | **Normal renal function**  **CrCl ≥ 90 mL/min** | | **Mild renal impairment**  **CrCl 60–89 mL/min** | | **Moderate renal impairment**  **CrCl 30–59 mL/min** | |
| --- | --- | --- | --- | --- | --- | --- |
|  | **FTD/TPI**  **(n = 31)** | **Placebo**  **(n = 19)** | **FTD/TPI**  **(n = 71)** | **Placebo**  **(n = 31)** | **FTD/TPI**  **(n = 49)** | **Placebo**  **(n = 21)** |
| **OS,** months |  |  |  |  |  |  |
| Median (95% CI) | 5.7 (3.9–9.0) | 3.9 (2.1–6.3) | 6.2 (4.5–11.0) | 6.3 (4.9–11.0) | 6.3 (4.6–7.5) | 4.1 (2.1–9.4) |
| HR (95% CI) | 0.71 (0.33–1.57) | | 0.86 (0.50–1.49) | | 0.79 (0.42–1.48) | |
| **OS rates,** % |  |  |  |  |  |  |
| 6-month (95% CI) | 45 (27–62) | 34 (14–56) | 50 (38–61) | 53 (33–69) | 54 (39–68) | 48 (26–67) |
| **PFS,** months |  |  |  |  |  |  |
| Median (95% CI) | 2.1 (1.9–3.7) | 1.8 (1.1–1.9) | 2.1 (1.9–3.5) | 1.9 (1.7–1.9) | 2.8 (1.9–3.7) | 1.7 (1.0–2.0) |
| HR (95% CI) | 0.31 (0.14–0.66) | | 0.49 (0.29–0.81) | | 0.60 (0.33–1.10) | |
| **PFS rates,** % |  |  |  |  |  |  |
| 4-month (95% CI) | 25 (11–41) | 0 | 30 (20–42) | 5 (<1–18) | 30 (17–43) | 14 (4–32) |

*CI* confidence interval; *CrCl* creatinine clearance; *FTD/TPI* trifluridine/tipiracil; *HR* hazard ratio; *OS* overall survival; *PFS* progression-free survival.

^a^As-treated population; does not include 3 patients with severe renal impairment and 3 patients whose renal status was missing.

# Table S4 Summary of grade ≥ 3 adverse events^a^

|  | **Number of patients (%)** | | | | | |
| --- | --- | --- | --- | --- | --- | --- |
|  | **< 65 years** | | **≥ 65 years** | | **≥ 75 years** | |
|  | **FTD/TPI**  **(n = 182)** | **Placebo**  **(n = 96)** | **FTD/TPI**  **(n = 153)** | **Placebo (n = 72)** | **FTD/TPI**  **(n = 50)** | **Placebo**  **(n = 17)** |
| **Grade ≥ 3 AEs of any cause** | 145 (80) | 60 (62) | 122 (80) | 37 (51) | 40 (80) | 9 (53) |
| Grade ≥ 3 treatment-related AEs | 89 (49) | 14 (15) | 87 (57) | 8 (11) | 28 (56) | 2 (12) |
| **Grade ≥ 3 AEs of any cause in ≥10% of patients in any group** | | | | | | |
| Hematologic |  |  |  |  |  |  |
| Neutropenia^b^ | 53 (29) | 0 | 61 (40) | 0 | 20 (40) | 0 |
| Anemia^c^ | 36 (20) | 7 (7) | 28 (18) | 6 (8) | 11 (22) | 3 (18) |
| Leukopenia^d^ | 17 (9) | 0 | 14 (9) | 0 | 7 (14) | 0 |
| Gastrointestinal |  |  |  |  |  |  |
| Vomiting | 10 (5) | 3 (3) | 2 (1) | 0 | 0 | 0 |
| Abdominal pain | 11 (6) | 10 (10) | 3 (2) | 5 (7) | 0 | 2 (12) |
| Ascites | 9 (5) | 7 (7) | 3 (2) | 4 (6) | 2 (4) | 1 (6) |
| Gastric hemorrhage | 3 (2) | 1 (1) | 0 | 3 (4) | 0 | 2 (12) |
| Other AEs |  |  |  |  |  |  |
| Asthenia | 8 (4) | 7 (7) | 8 (5) | 4 (6) | 3 (6) | 2 (12) |
| Decreased appetite | 15 (8) | 7 (7) | 14 (9) | 4 (6) | 5 (10) | 2 (12) |
| Fatigue | 12 (7) | 7 (7) | 11 (7) | 3 (4) | 4 (8) | 2 (12) |
| General physical health deterioration | 15 (8) | 8 (8) | 7 (5) | 7 (10) | 1 (2) | 1 (6) |

*AE* adverse event; *FTD/TPI* trifluridine/tipiracil.

^a^As-treated population.

^b^Includes decreased neutrophil count.

^c^Includes decreased hemoglobin.

^d^Includes decreased white blood cell count.

# Table S5 Summary of safety by renal function status in patients aged ≥ 65 years^a^

|  | **Number of patients (%)** | | | | | |
| --- | --- | --- | --- | --- | --- | --- |
|  | **Normal renal function**  **CrCl ≥ 90 mL/min** | | **Mild renal impairment**  **CrCl 60–89 mL/min** | | **Moderate renal impairment**  **CrCl 30–59 mL/min** | |
|  | **FTD/TPI**  **(n = 31)** | **Placebo**  **(n = 19)** | **FTD/TPI**  **(n = 71)** | **Placebo**  **(n = 31)** | **FTD/TPI**  **(n = 49)** | **Placebo**  **(n = 21)** |
| **AEs of any cause** | 30 (97) | 18 (95) | 68 (96) | 26 (84) | 47 (96) | 21 (100) |
| Grade ≥ 3 | 23 (74) | 12 (63) | 59 (83) | 14 (45) | 38 (78) | 10 (48) |
| **Treatment-related AEs** | 25 (81) | 8 (42) | 58 (82) | 18 (58) | 37 (76) | 10 (48) |
| Grade ≥ 3 | 14 (45) | 3 (16) | 43 (61) | 3 (10) | 28 (57) | 2 (10) |
| **Actions taken because of AEs of any cause** | | | | | | |
| Dosing modification | 19 (61) | 5 (26) | 42 (59) | 5 (16) | 31 (63) | 6 (29) |
| Discontinuation | 4 (13) | 2 (11) | 6 (8) | 4 (13) | 8 (16) | 3 (14) |
| **Most common AEs of any cause and grade (≥ 15% of patients)** | | | | | | |
| Hematologic |  |  |  |  |  |  |
| Neutropenia^b^ | 16 (52) | 0 | 43 (61) | 1 (3) | 25 (51) | 2 (10) |
| Anemia^c^ | 12 (39) | 1 (5) | 26 (37) | 6 (19) | 31 (63) | 4 (19) |
| Leukopenia^d^ | 5 (16) | 0 | 14 (20) | 0 | 15 (31) | 1 (5) |
| Thrombocytopenia^e^ | 4 (13) | 0 | 14 (20) | 1 (3) | 10 (20) | 3 (14) |
| Gastrointestinal |  |  |  |  |  |  |
| Abdominal pain | 6 (19) | 2 (11) | 12 (17) | 5 (16) | 6 (12) | 5 (24) |
| Vomiting | 6 (19) | 5 (26) | 13 (18) | 6 (19) | 11 (22) | 3 (14) |
| Nausea | 5 (16) | 3 (16) | 21 (30) | 6 (19) | 13 (27) | 6 (29) |
| Diarrhea | 3 (10) | 3 (16) | 19 (27) | 5 (16) | 13 (27) | 3 (14) |
| Constipation | 4 (13) | 2 (11) | 8 (11) | 4 (13) | 9 (18) | 2 (10) |
| Upper abdominal pain | 2 (6) | 3 (16) | 1 (1) | 1 (3) | 3 (6) | 1 (5) |
| Other AEs |  |  |  |  |  |  |
| Decreased appetite | 12 (39) | 4 (21) | 25 (35) | 8 (26) | 19 (39) | 5 (24) |
| Fatigue | 9 (29) | 5 (26) | 14 (20) | 11 (36) | 15 (31) | 0 |
| Asthenia | 5 (16) | 6 (32) | 17 (24) | 7 (23) | 12 (24) | 4 (19) |
| Increased blood alkaline phosphatase | 3 (10) | 2 (11) | 6 (9) | 6 (19) | 5 (10) | 1 (5) |

*AE* adverse event; *CrCl* creatinine clearance; *FTD/TPI* trifluridine/tipiracil.

^a^As-treated population.

^b^Includes decreased neutrophil count.

^c^Includes decreased hemoglobin.

^d^Includes decreased leukocyte count.

^e^Includes decreased platelet count.

**Table S6.** Time to deterioration by ≥ 5 points in EORTC QLQ-C30 global health status scores by age^a^

|  |  | | | | | |
| --- | --- | --- | --- | --- | --- | --- |
|  | **< 65 years** | | **≥ 65 years** | | **≥ 75 years** | |
|  | **FTD/TPI**  **(n = 159)** | **Placebo**  **(n = 76)** | **FTD/TPI**  **(n = 129)** | **Placebo (n = 54)** | **FTD/TPI**  **(n = 42)** | **Placebo**  **(n = 12)** |
| Median time to deterioration (months) | 2.3 | 1.9 | 3.3 | NE | 3.3 | 3.8 |
| 95% CI | 2.0–2.6 | 1.2–4.6 | 2.4–4.9 | 1.9–NE | 2.1–4.8 | 1.0–NE |
| HR (95% CI) | 1.30 (0.70–2.43) | | 1.64 (0.80–3.36) | | 0.78 (0.07–8.88) | |

*CI* confidence interval*; EORTC QLQ-C30,* European Organization for Research and Treatment of Cancer Quality of Life questionnaire; *FTD/TPI* trifluridine/tipiracil; *HR* hazard ratio; *NE*, not estimable.

^a^Includes patients with QLQ-C30 assessments completed at baseline and at least one follow-up visit.

# Fig. S1 Incidences of most common adverse events in FTD/TPI-treated patients by age.

*FTD/TPI* trifluridine/tipiracil.

#
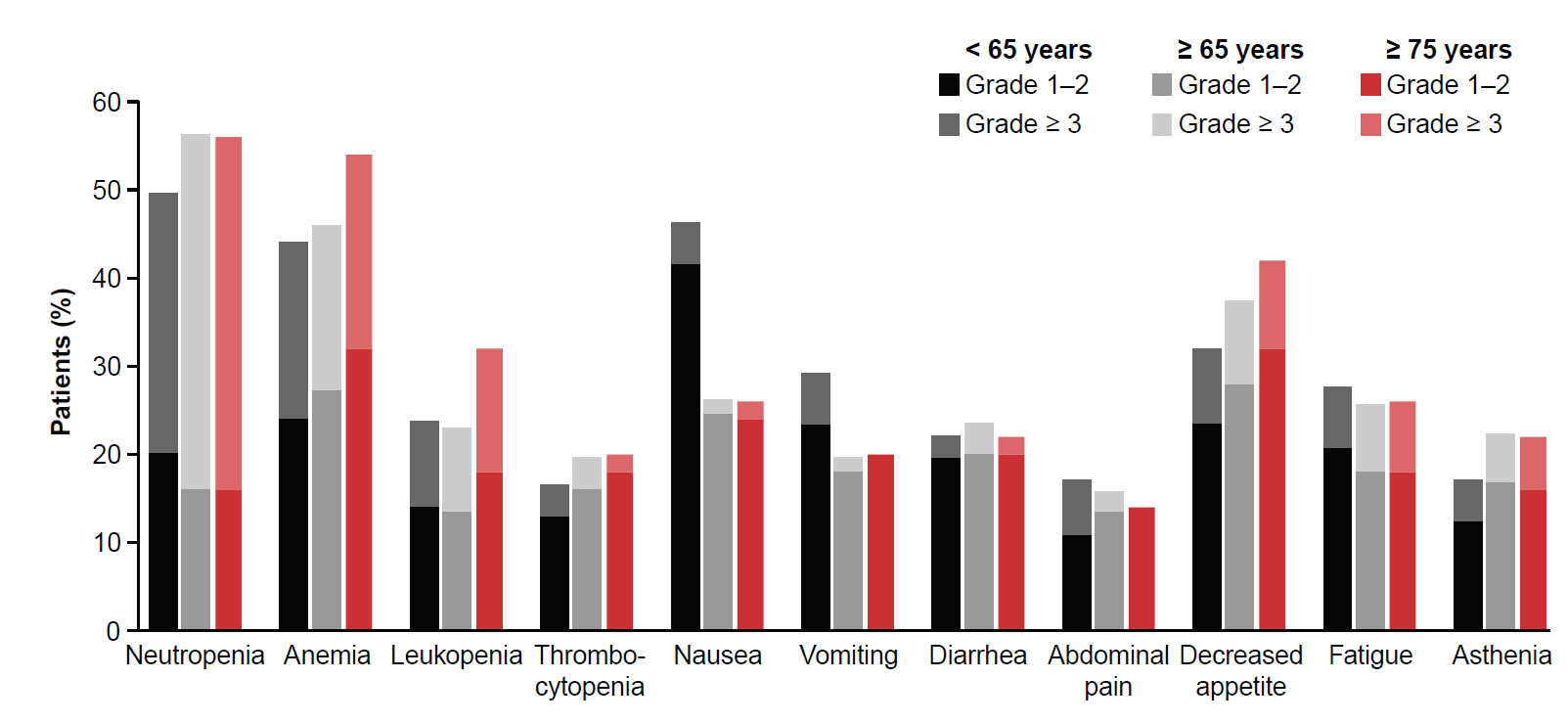


**Fig. S2** Odds ratio^a^ of common adverse events in patients aged (a) < 65 years, (b) ≥ 65 years, and (c) ≥ 75 years.

^a^Odds ratio > 1 indicates a greater treatment effect (FTD/TPI vs placebo).

*FTD/TPI* trifluridine/tipiracil.

**
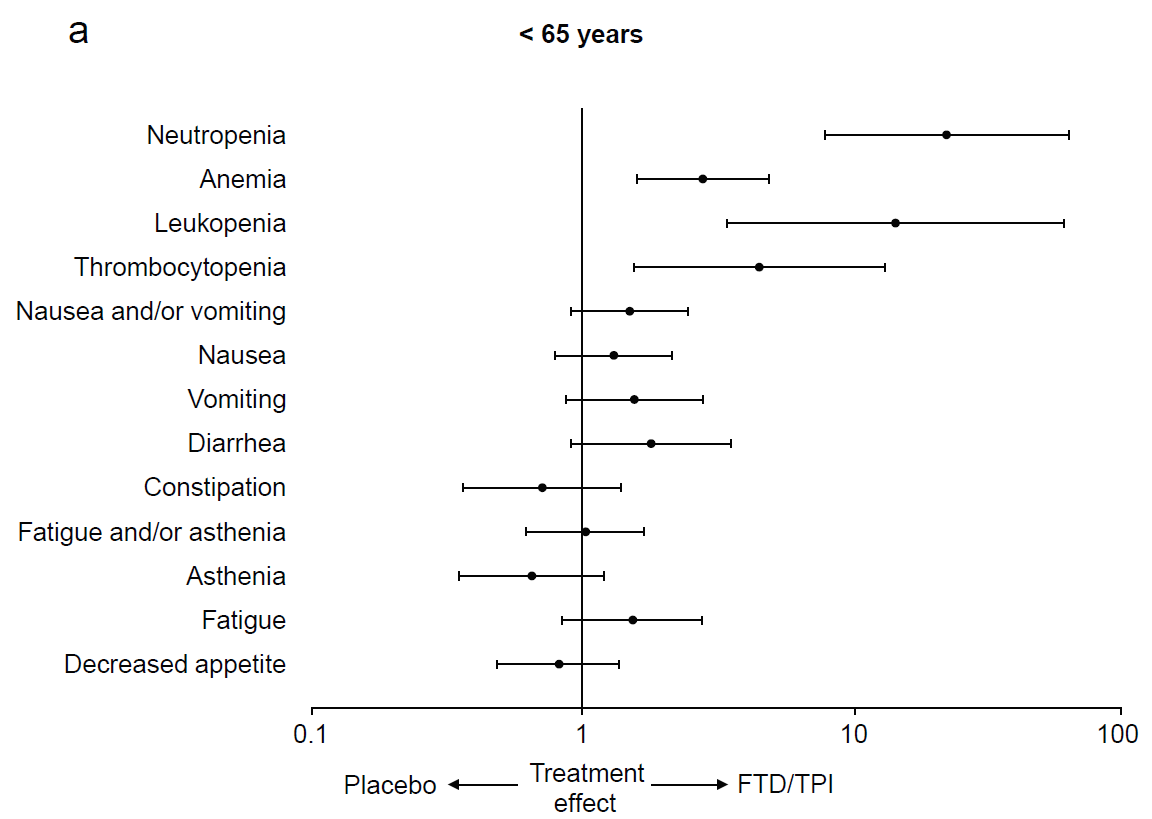
**

**
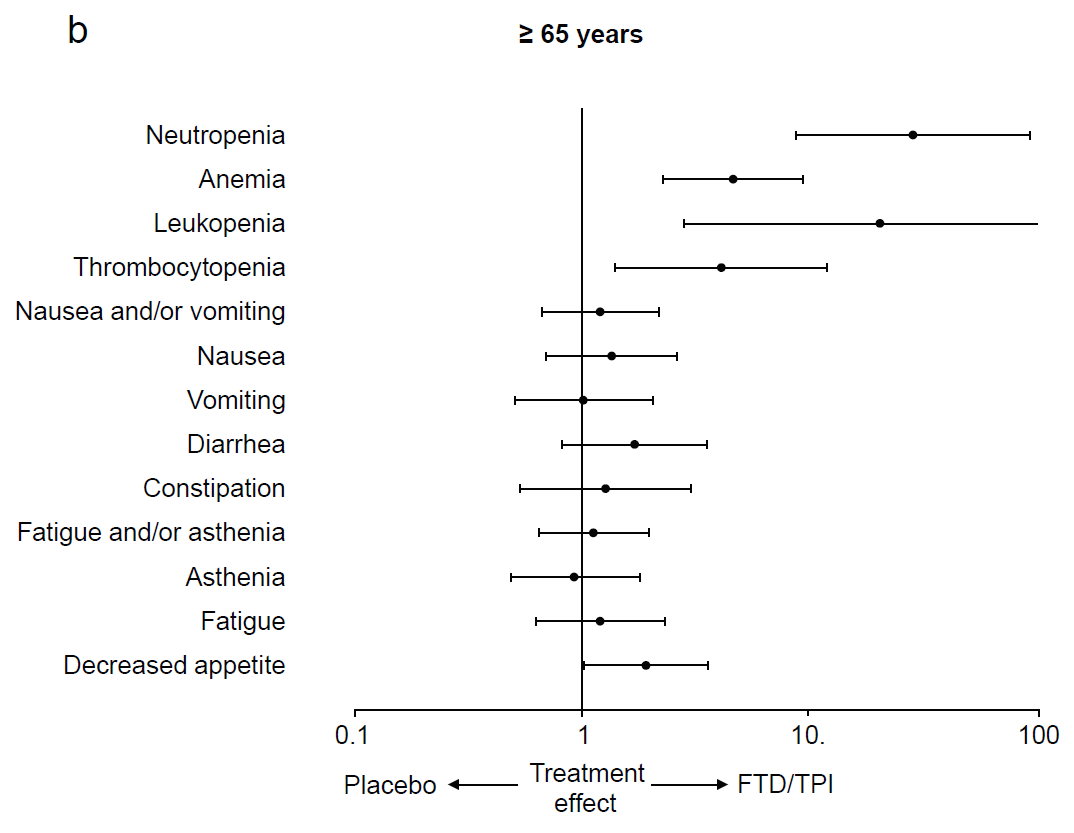
**


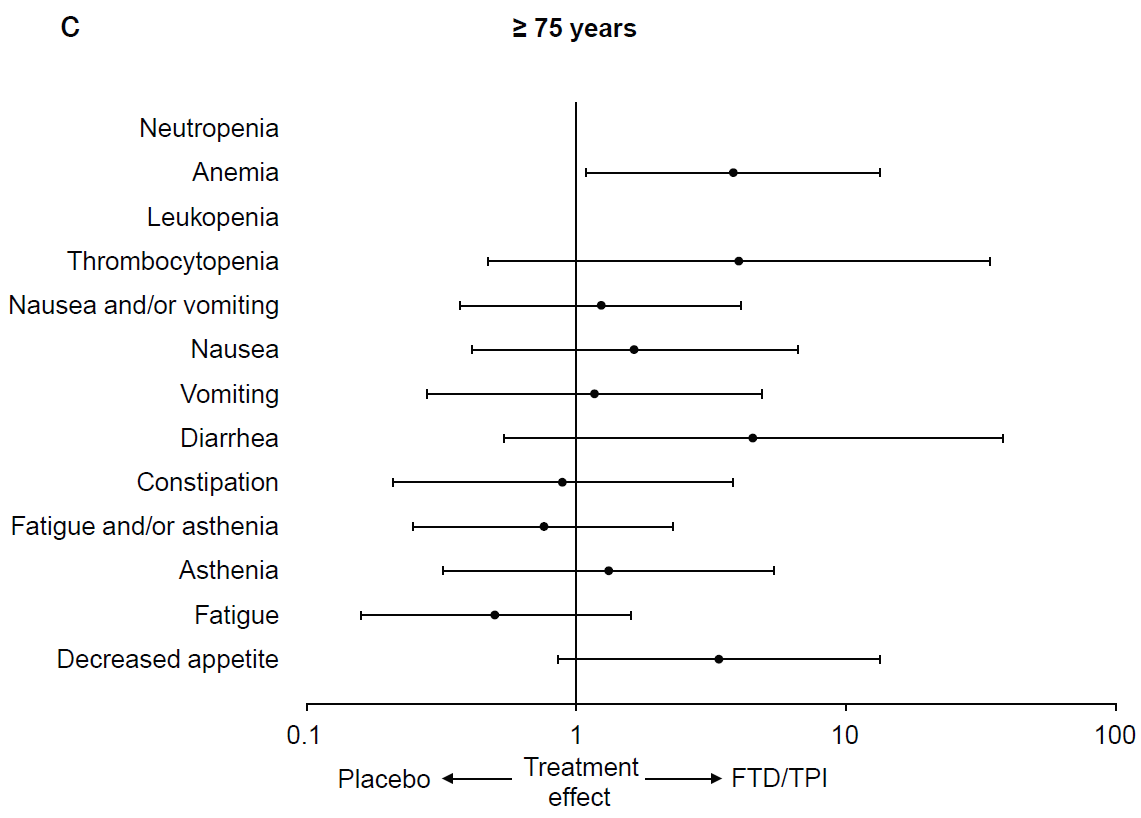

Supplement: Supplementary file 1 — Supplementary file1 (DOCX 283 KB) [file 10120_2021_1271_MOESM1_ESM.docx]
